# Commending rather than condemning: Moral elevation and stigma for male veterans with military sexual trauma

**DOI:** 10.1186/s40359-022-01002-4

**Published:** 2022-12-06

**Authors:** Gracie Staley, Ana Clara Vieira Zaidan, Katrina Henley, Lucas G. Childers, Ray Daniel, Sean A. Lauderdale, Adam P. McGuire

**Affiliations:** 1 Department of Psychology and Counseling, The University of Texas at Tyler, Tyler, TX USA; 2 Department of Psychology and Sociology, Texas A&M University Corpus Christi, Corpus Christi, TX USA; 3 VISN 17 Center of Excellence for Research on Returning War Veterans, Waco, TX USA; 4 Central Texas Veterans Health Care System, Temple, TX USA; 5 Department of Psychology and Special Education, Texas A&M University Commerce, TX Commerce, USA

**Keywords:** Moral elevation, Stigma, Military sexual assault, Veterans

## Abstract

**Background:**

Using an experimental study, we examined the link between state moral elevation and stigmatic beliefs surrounding male veterans with military sexual trauma (MST).

**Methods:**

Undergraduate students were presented with a video or written narrative of a male veteran self-disclosing how they struggled with and overcame MST (*n* = 292). Participants completed measures regarding trait and demographic characteristics at baseline, then measures immediately after the disclosure stimulus to assess immediate elevation and stigma-related reactions.

**Results:**

Results suggest state-level elevation in response to a veteran self-disclosing their experience with MST was negatively correlated with harmful stigmatic beliefs about MST. A greater predisposition to experience elevation and PTSD symptoms were linked with stronger elevation responses to the stimulus.

**Conclusion:**

Findings support the need for further exploration of elevation and its potential to impact public stigma for male veterans with MST.

## Introduction

Broadly, stigma is based on a belief that a person or group with a specific trait is considered deviant, which is associated with negative stereotypes or perceptions of that person [1]. Stigma surrounding mental health in particular can include a wide range of negative stereotypes such as believing someone has personal responsibility for experiencing mental health issues, feelings of pity, anger, or fear toward that person, believing one should withhold help or avoid those with distress, and support for segregation and coercion [2]. Unfortunately, there is a substantial amount of stigma related to veteran mental health problems, particularly for male sexual assault [3]. Additional research is needed to understand novel pathways that could positively impact the public stigma surrounding military sexual trauma, which some veterans are forced to confront. Previous studies suggest one potentially relevant construct is moral elevation—an emotion experienced after witnessing another person perform a virtuous act [4]. This experimental study aims to investigate the link between moral elevation and stigma for male veterans who experienced sexual assault.

## Military sexual trauma and public stigma

The military, along with most workplaces in the United States (US), is not immune from sexual harassment or assault. The rate of sexual assault experienced in the military is believed to mirror the rate in civilians with reports ranging from 22 to 33% for women and 1 to 12% for men [5]. The actual rate of military sexual trauma (MST) is unknown for several reasons including potential biases, stigma, or fears associated with reporting assaults; however, it is estimated that 15.7% of current military personnel and veterans have reported MST during their time in service [6]. Despite the myth that men are rarely sexually assaulted, several nationwide surveys found that men are assaulted at a similar rate to women [7]. This is the same for the military, given that approximately 50% of MST survivors are male [8]. Following an assault, experiences of MST can lead to severe consequences including significant distress, posttraumatic stress disorder (PTSD), and potential functional impairment [9], Thus, many veterans are forced to confront the serious problem of experiencing MST and subsequent negative outcomes.

In the context of military service and post-deployment, previous work has established there are stigmatic beliefs surrounding mental health concerns and treatment-seeking in general [10, 11]. However, public stigma for MST in male veterans is particularly salient given societal expectations for gender and sexuality, along with myths about who can be raped or assaulted. Male rape myths can include beliefs that *real* men cannot be raped or can defend themselves to prevent it from happening, along with beliefs regarding homosexuality such as only gay men are raped or that rape survivors become gay [12]. Researchers have proposed that these societal expectations or stigma for assault survivors is an important source of psychological distress. For example, a qualitative study with a civilian sample found that young men who experienced sexual assault reported feeling shame, embarrassment, disempowerment, and emasculation, which was fueled by stigmatic beliefs regarding male victimization [13]. Findings suggest these feelings contributed to low rates of disclosure and an underreporting of assault in this sample. The issue of disclosure hesitancy is further compounded for service members given that both men and women may not report an assault out of fear of social retaliation in the military [14]. A separate study of male veterans also reported frequent negative reactions to disclosure that were harmful, whereas positive reactions reportedly aided the recovery process [15]; further highlighting that public perceptions of MST survivors can play an important role. Overall, public stigma surrounding this topic is particularly problematic insofar as it could contribute to reduced treatment seeking and support for male veteran with MST who are in need of additional care. Therefore, efforts to address consequences of stigma should consider novel pathways to reduce public stigma. One potential pathway that could help address this concern is moral elevation.

## Moral elevation and links with stigma

Moral elevation (hereafter, elevation) is a positive emotion experienced after witnessing someone perform a virtuous or altruistic act [4, 16–18]. Elevation is further distinguished from other positive psychology constructs like admiration or gratitude by its emotional responses (feeling *inspired*, *uplifted*, *moved*), physical responses (*tears in eyes*, *lump in throat*) and subsequent motives to perform similar virtuous acts or engage in prosocial behavior [19–23]. Because the motives associated with elevation are antithetical to the negative attitudes and behaviors of stigma, it follows that eliciting elevation might be a useful counteracting agent to stigmatic beliefs.

Accordingly, elevation has been linked with a wide range of benefits and positive correlates including reduced stigma or prejudice against marginalized groups. For example, one study found participants who were exposed to elevating videos compared to a control condition reported reduced implicit and explicit prejudice toward gay men [24]. There is also evidence that inducing elevation is associated with more prosocial behaviors towards an outgroup or a minoritized group by socially dominant groups [25]. However, there are mixed findings that suggest elevation may not lead to significant differences in prejudice, such as reducing homophobia [26]. Therefore, further research is needed to better understand the link between elevation and stigma, particularly for stigmatic beliefs surrounding MST in male veterans. The authors are unaware of previous studies that have formally tested the potential impact of elevation on this specific stigmatic belief or examined the role of elevation in stigma towards veterans in general.

If elevation does in fact reduce stigma for MST, it would be important to expand our understanding of how to elicit this emotion and identify any characteristics that might predispose someone to feel elevated in this context. One approach that could possibly elicit elevation within the context of MST is to present a story about a male veteran disclosing his experience with assault. Given the societal expectations surrounding sexual assault survivors [12] and previous findings that highlight the hesitancy and fear associated with self-disclosure [13, 15], a story of a male veteran who openly shares his experiences with MST could be perceived as a remarkable act of courage. A story that also includes a description of his path to recovery and engagement with treatment could also demonstrate significant perseverance and hope. In terms of practical steps for using such a story to elicit elevation, past work has demonstrated elevation can be induced in experimental designs with different approaches such as viewing videos that display virtuous acts [16, 20, 21, 27], reading stories about virtuous behavior [25, 28], or using a recall technique [29, 30]. Yet few studies have directly examined the impact of induction method types to determine if there are significant differences in elevation responses between viewing videos versus reading written narratives, for example. Thus, attempts to elicit elevation using a story of MST self-disclosure should examine both video and written narrative formats to determine if either format is more effective for this subject.

In addition to the content and format of potential elevation stimuli, it is important to understand predisposing characteristics that might indicate who is likely to report a stronger elevation response to self-disclosure of MST. Although no known studies have examined relevant factors for eliciting elevation around this specific topic, there is some evidence that the capacity for inspiration or elevation is higher when the witness shares similarities with the target exemplar (i.e., person demonstrating virtuous act) or perceives that exemplar as relatable [31, 32]. In this case, the gender of the witness could play an important role since men might perceive the story differently in the context of societal beliefs or pressures regarding masculinity. Another important set of factors could be whether or not witnesses endorse PTSD symptoms themselves and whether they know someone who is a survivor of sexual assault. Experiencing PTSD symptoms could make the exemplar more relatable, and having endured those symptoms or known someone who suffered from a similar trauma might lead the viewer to have a greater appreciation for the level of courage, perseverance, or hope that is being demonstrated by an exemplar (thus, leading to higher elevation). Age might be another relevant characteristic to consider. Significant differences in age between the witness and the exemplar could be a barrier to perceived relatability and might negatively impact the elevation response. Additionally, previous studies found that age was positively correlated with elevation responses [33].

Beyond shared characteristics, innate beliefs about male rape myths could play an important role in elevation responses. As previously noted, existing myths include the belief that men cannot be truly raped, men who were raped are gay, or will become gay. Although no studies have directly examined the relation between elevation responses and male rape myths, it is plausible that if present, these biases and reductive perspectives could present a barrier to feeling inspired by a male veteran who discloses his experience with MST.

On the other hand, some trait characteristics might positively impact state elevation. The most relevant would be a greater predisposition to experience elevation, which could be described as trait-like elevation or the tendency to engage with moral beauty [34]. Past work found that people who report a greater tendency to experience elevation in response to acts of moral beauty or virtue endorse higher levels of state elevation following relevant stimuli [27, 35]. Another potential predictor could be dispositional empathy or the tendency to express empathy for others, which may impact a witness’s perception of the exemplar’s moral character [36]; thus, contributing to positive assessments of a male veteran who experienced MST. Few studies have tested the direct relation between *trait* empathy and *state* elevation; however, there is preliminary support that empathy and elevation are positively correlated at the trait-level [27] and state-level [37], separately.

State elevation in response to a male veteran who self-discloses their history with MST could be impacted by a wide range of factors including shared characteristics or experiences, predispositions to respond to moral acts, and beliefs surrounding male rape myths. Predictors of state elevation in this context are largely unknown, yet it is important to identify who is likely to endorse positive responses, especially if elevation is linked with lower levels of stigma for MST.

## Current study

The purpose of this study is to examine moral elevation and stigmatic beliefs about MST as potential responses to witnessing a veteran self-disclose their experience with MST. Specifically, this study included three aims. First, we examined elevation responses to the disclosure by inspecting descriptive statistics and assessed whether elevation responses differed based on the format of the disclosure (video versus transcribed formats). Next, we explored potential predictors of elevation responses, including theoretically relevant traits and characteristics that align with the veteran featured in the disclosure. Aims one and two were considered exploratory and did not include a priori hypotheses. Lastly, we assessed if higher elevation following a disclosure of MST was correlated with lower levels of stigma across nine domains. We hypothesized a negative correlation between elevation and harmful stigma reported.

## Method

### Participants

Participants were recruited from an undergraduate subject pool of psychology students from a university in the Southern United States. The initial sample size recruited was 323 participants. Seven participants were removed because they did not consent, 23 participants were excluded because they failed an attention screen, and 1 participant was removed because they had a large portion of missing data (29%). The final sample included 292 participants (*M*_*age*_ = 22.85, *SD* = 7.47; 82.53% female) with 46.6% identifying as White, 29.5% as Black, 22.6% as Hispanic, 2.7% as American Indian or Alaska Native, 2.4% as Asian or Asian American, 0.7% Native Hawaiian or Pacific Islander, and 3.1% as Other.

### Procedure

Interested students followed a survey link that led to a brief summary of the study and then provided online consent. Within the survey, first, participants were presented with demographic questions that asked for their age, race/ethnicity, and gender. Participants were also asked whether they know someone who experienced sexual assault (yes/no). Next, participants were given a set of randomized baseline questionnaires that assessed traits and history relevant to MST. Following the baseline measures, all participants were presented with a vignette about a male veteran who experienced a sexual assault in the military and who sought treatment after struggling to disclose that experience for a period of time. Participants were randomly assigned to either (a) watch a video of that male veteran discussing his experience or (b) read a transcript of that same video describing the experiences of the same veteran. Lastly, the participants completed follow-up questionnaires immediately after the vignette that assessed their responses to the vignette, including a state elevation measure and a questionnaire about stigma toward that specific veteran. An attention screen (“What branch was the veteran from?”) was also included for all participants within the final set of questionnaires to verify the participant watched or read the vignette. Participants with incorrect responses were excluded from data analysis. All participants consented to participate and study procedures were approved by the local Institutional Review Board.

### Measures

#### Empathy

The Toronto Empathy Questionnaire [38] measured trait-level empathy. Participants rated the 16 items on a 0 (*never*) to 4 (*always*) scale. Sample items include “It upsets me to see someone being treated disrespectfully” and “I enjoy making other people feel better.” All items were summed to create a total score ranging between 0 and 64 with higher scores representing greater empathy (α = .89 [95% CI: .87, .91]).

#### Trait Moral Elevation

The Engagement with Beauty Scale [34] is designed to assess trait-like tendencies to feel moved or inspired by natural, artistic, and moral beauty. The moral beauty subscale was used to assess predisposition to experience moral elevation (i.e., trait elevation). Participants rated six items on a scale from 1 (*very unlike me*) to 7 (*very like me*). Sample items include “I notice moral beauty in human beings” and “When perceiving an act of moral beauty, I find that I desire to become a better person.” The items were summed to create a subscale score ranging between 6 and 42 with higher scores representing higher trait elevation (α = .87 [.85, .89]).

#### PTSD symptoms

The Posttraumatic Diagnostic Scale [39] is a 17-item self-report questionnaire that was used to assess PTSD symptoms. Participants were asked to rate the extent they experienced items on a scale from 0 (*not at all*) to 3 (*3–5 or more time a week/very much/almost always*). Sample items include “Having bad dreams or nightmares about the traumatic event” and “Trying not to think or talk about the traumatic event.” Due to an error during survey construction, one item was missing in the survey that was administered to all participants (item 7: avoiding activities, people, and places). Therefore, we calculated the mean item score for the remaining 16 items as the total score ranging between 0 and 3 with higher scores representing more severe PTSD symptoms. Although this deviation from standard administration is a study limitation, internal consistency was still high (α = .94 [.93, .95]).

#### Male rape myth beliefs

The Male Rape Myth Scale [40, 41] assessed false and stereotypical beliefs about male rape. Participants rated 22 items on a scale from 1 (*strongly disagree*) to 6 (*strongly agree*). Sample items include “Any healthy man can successfully resist a rapist if he really wants to” and “A man who has been raped has lost his manhood.” All items were summed to create a total score ranging between 22 and 132 with higher scores indicating greater acceptance of male rape myths (α = .92 [.91, .93]).

#### Stigma response

The Attributions Questionnaire-27 [2] measured nine stereotypes about the specific veteran presented in the randomized vignette. Immediately after reviewing the vignette, participants rated the extent to which they agreed with 27 items, as they relate to the veteran self-disclosing MST, on a scale from 1 (*none at all*) to 9 (*very much*). This questionnaire was designed to assess nine factors that pertain to public attitudes, emotional affect, and behaviors surrounding stigma for mental health [42]. Accordingly, nine subscale scores were created by summing the three items for each factor, ranging between 3 and 27, with higher scores representing stronger endorsement of that stereotype. Subscales included stereotypes related to anger (e.g., “I would feel aggravated by the veteran.”), blame (e.g., “I would think it was the veteran’s own fault that he is in the present condition.”), pity (e.g., “I would feel pity for the veteran.”), help (e.g., “I would be willing to talk to the veteran about his problems.”), dangerousness (e.g., “I would feel unsafe around the veteran.”), fear (e.g., “The veteran would terrify me.”), avoidance (e.g., “If I were an employer, I would interview the veteran for a job.”; reverse-scored), segregation (e.g., “I think the veteran poses a risk to his neighbors unless he is hospitalized.”), and coercion (e.g., “If I were in charge of the veteran’s treatment, I would force him to take his medication.”). Internal consistency for subscales in this study were mostly adequate (see Table 2) with the exception of blame (α = .61 [.54, .69]) and coercion (α = .47 [.37, .57]).

#### State moral elevation response

The State Moral Elevation Scale [35] measured state-level elevation in response to reviewing the veteran vignette. Participants rated the extent they experienced nine items using a scale from 0 (*not at all*) to 4 (*extremely*). Sample items include “Somehow lifted up or in touch with the better parts of myself” and “Motivated to live in a nobler or virtuous way.” All items were summed to create a total score ranging between 0 and 36 with higher scores representing greater elevation experienced (α = .89 [.87, .91]).

### Data analysis

All data management and analyses were conducted with R [43]. First, to examine whether elevation responses differed between the video versus transcript condition, we calculated an independent samples *t*-test with the t-test function from the base R stats package; Cohen’s *d* was calculated with the psych package. We interpreted *d* > 0.20, *d* > 0.50, and *d* > 0.80 as small, medium, and large effects [44]. For the second aim that examined baseline characteristics as predictors of the state elevation response, we used the lm function from the base R stats package to fit a linear regression model that included the following predictors, standardized: gender, age, trait elevation, trait empathy, PTSD symptoms, male rape myth beliefs, and whether or not they know someone who is a sexual assault survivor (yes = 1, no = 0). Lastly, to test our hypothesis that elevation is negatively correlated with stigma, correlations were calculated with the psych package [45].

## Results

First, an independent samples *t*-test was performed to compare the mean level of state elevation experienced following the video vignette (*M* = 28.08, *SD* = 7.83, *n* = 137) and transcript vignette (*M* = 28.68, *SD* = 7.50, *n* = 155). Results indicated elevation was not statistically different between the two groups with no meaningful effect size (*t* = -0.66, *p* = .509, *d* = 0.08).

Regarding baseline characteristics, results from the linear regression model indicated the only significant predictors of state elevation experienced after the vignette were trait elevation and PTSD symptoms (see Table 1). Gender, age, empathy, male rape myth beliefs, and knowing a sexual assault survivor were all nonsignificant.

**Table 1:** Baseline characteristics as predictors of state elevation response to male MST disclosure

Variable	β (*SE*)	*p*
Gender	− 0.03 (0.15)	.866
Age	− 0.01 (0.01)	.062
Trait elevation	**0.34 (0.07)**	**< .001**
Trait empathy	0.08 (0.07)	.211
PTSD symptoms	**0.17 (0.05)**	**.002**
Male rape myth beliefs	− 0.02 (0.06)	.688
Known sexual assault survivor	− 0.19 (0.11)	.101

Model summary statistics: *F*_(7, 283)_ = 8.68, *p* < .001, *R*^*2*^ = .18. Boldface indicates statistical significance at *p* < .05.

Bivariate correlations between state elevation and separate stigma domains, along with descriptive statistics for domain subscales are described in Table 2. Elevation experienced following the MST vignette demonstrated a small, positive correlation with greater pity, and greater willingness to help, whereas there was a small, negative correlation with avoidance. All other correlations with the remaining stigma domains were not statistically significant with correlation sizes below 0.10.

**Table 2:** Descriptive statistics and bivariate correlations between state moral elevation and stigma domains

Covariate	*M*	*SD*	Range	*r* (95% CI)	*p*	α (95% CI)
Anger	5.22	4.38	3–27	.05 [− .07, .16]	.401	.88 [.85, .90]
Blame	8.34	5.09	3–27	.05 [− .07, .16]	.439	.61 [.54, .69]
Pity	**19.40**	**6.10**	**3–27**	**.23 [.12, .34]**	**.000**	**.77 [.73, .82]**
Help	**21.26**	**6.71**	**3–27**	**.20 [.09, .31]**	**.001**	**.92 [.91, .94]**
Dangerousness	5.79	4.82	3–27	.03 [− .09, .14]	.646	.90 [.89, .92]
Fear	5.74	4.68	3–27	.05 [− .07, .16]	.435	.88 [.85, .90]
Avoidance	**9.71**	**6.85**	**3–27**	**− .17 [-.27, − .05]**	**.005**	**.88 [.85, .90]**
Segregation	6.01	4.82	3–27	.04 [− .07, .16]	.477	.89 [.87, .91]
Coercion	10.41	4.87	3–23	.06 [− .06, .17]	.315	.47 [.37, .57]

Boldface indicates *p* < .05.

## Discussion

The purpose of this study was to examine moral elevation and stigmatic beliefs as responses to a male veteran who disclosed his experience with MST. Results for exploratory aims indicated there was no significant difference in state elevation between participants who were presented with a video versus written narrative format of the disclosure. Additionally, results identified trait elevation and PTSD symptoms as baseline characteristics associated with higher state elevation responses. Lastly, we found evidence that partially supported our hypothesis that higher elevation after witnessing someone disclose MST would be correlated with lower negative stigma for that person.

### Comparing moral elevation responses to video and written formats

First, there was no significant difference in elevation responses after watching a video or reading a transcript of that exact same video. These results could offer preliminary support to studies that have used either approach to elicit elevation in past work. One potential reason neither approach was superior could be that elevation is posited to be, in part, a cognitive experience that involves making a specific attribution about an observed act [34]. In this case, it appears that one stimulus type (i.e., video or transcript) did not elicit more elevation than the other when the content was the same.

Notably, our study only compared one vignette focusing on MST and treatment-seeking behavior across both induction methods. Perhaps, demonstrations of moral beauty with different stories or protagonist characteristics might elicit elevation more easily through either method. Future research should examine this further by assessing potential differences in elicited elevation by format when using a wide range of stories or persons (i.e., extending beyond stories about MST). If this approach is to be used to target public stigma, future work should also examine other potential formats of elevation induction methods (e.g., audio), which may expand options for conducting elevation research more broadly.

### Predictors of moral elevation response to MST disclosure

Findings suggest trait elevation and PTSD symptoms were significantly associated with having a higher elevation response to the MST vignette. This is consistent with previous studies that found trait-like elevation is highly correlated with a state elevation response to witnessing moral beauty [27, 34]. However, to our knowledge, no known studies have examined current PTSD symptoms as a predictor of state elevation. Perhaps the narrative of this particular veteran’s trauma and recovery journey elicits more elevation in people with PTSD symptoms because they have a greater appreciation for the challenges that veteran faced, which would also be consistent with previous work that indicates similarities with the exemplar is a predictor of stronger responses [31, 32]. If experiencing state elevation can lead to desirable outcomes or correlates as indicated in previous research, results could suggest these types of narratives and disclosures might also benefit those who have experienced MST.

These findings are also important insofar as they may inform who should be targeted for attempts to elicit elevation in response to stigmatized topics, particularly within groups like veterans who are at risk for experiencing problems associated with MST. Creating opportunities for people to feel inspired by veterans self-disclosing a stigmatized experience of sexual assault, or mental health issues broadly, could be of interest to organizations that want to increase engagement for veterans with similar backgrounds (e.g., Veterans Affairs Medical Centers, Veteran Support Networks) or facilitate community engagement and support when veterans are transitioning to civilian life.

### Correlations between moral elevation and stigma domains

Results demonstrated that higher elevation was correlated with greater pity, greater willingness to help, and lower avoidance. These findings are somewhat consistent with other research that found elevation was linked with lower levels of public stigma, broadly defined. For example, Freeman et al. [25] demonstrated that inducing elevation using a video about “outside groups” could be used to reduce stigma and increased willingness to help outgroups. As previously noted, another study found evidence that elevation can reduce implicit and explicit sexual prejudice against gay men [24], aligning with the negative correlation found between elevation and avoidance-related stigma associated with MST. Although prejudice against homosexuality is different than stigma for males who were sexually assaulted regardless of sexuality, the two share similar prejudices or biases among the public [13, 46].

Notably, the correlations with elevation were positive for help and pity, whereas it was negatively correlated with avoidance. Corrigan et al. [2] define the help stereotype as, “the provision of assistance to people with mental illness” and the pity stereotype as, “sympathy because people are overcome by their illness.” The fact that elevation was positively correlated with these two domains is consistent with the body of evidence that suggests elevation is linked with strong urges or motives to connect with and help others [16, 33]. Additionally, researchers have demonstrated that elevation is an approach-oriented emotion [23], which is also consistent with the negative correlation for the avoidance stereotype described as, “stay away from people with mental illness” [2].

However, somewhat unexpectedly, results indicated the significant associations were limited to pity, help, and avoidance. State elevation was not correlated with other stereotypes, including anger, blame, fear, segregation, and coercion. These results suggest that elevation may only relate to specific features of public stigma, but it is unclear why that might be the case. More research is needed to replicate these findings and examine potential mechanisms or shared features that would explain the differences in associations across stigma types. For example, future studies could consider the attribution model of discrimination against mental health [2] and assess whether elevation induction can influence different stages of the model such as beliefs about personal responsibility for causing the condition, affective reactions, and behavioral responses. This approach may inform if and how elevation could be used as an intervention tool to target public stigma.

### Limitations and future directions

These findings should be interpreted while considering several limitations. First, participants were limited to undergraduate students at a university in the southern US, which is not representative of the overall population. It would be important to replicate these findings with a more diverse sample. Second, stigma was only measured after participants were exposed to the veteran narrative and we did not include a baseline assessment of stigma; therefore, it is unclear if significant correlations are a function of changes initiated by elevation or if they represent low levels of stigma at baseline. Future studies should aim to investigate the link between elevation and within-person changes in MST-related stigma. Third, we used a PTSD symptom measure that was based on the fourth version of the Diagnostic and Statistical Manual of Mental Disorders (DSM-IV-TR) rather than the recent fifth version (DSM-5). Additionally, this measure was missing one item in the final survey administration; therefore, future studies should aim to replicate the findings that PTSD symptoms are associated with elevation response to MST stimuli with updated measurement tools. Lastly, the results are primarily correlational and cannot establish a causal link between elevation and stigma. To fully understand how elevation is experienced and whether it can be used to target stigma, future research should assess causal relationships (e.g., the attribution model [2]) by using control conditions and explore changes in specific motivations or perceptions that might facilitate a reduction in stigmatic beliefs.

## Conclusion

In this study, there was preliminary evidence that moral elevation was correlated with stigmatic beliefs for MST. Additionally, some of the characteristics that were linked with greater elevation responses were trait elevation and PTSD symptoms—a shared experience with the veteran exemplar. Overall, these findings help expand the current understanding of the link between elevation and stigma within the context of a stigmatized issue for male veterans. Furthermore, results inform the use of elevating induction methods and suggest the need for further research to explore the use of elevation in efforts to impact public perceptions of male MST as a stigmatized issue.

## Data Availability

The datasets used and/or analyzed during the current study are available from the corresponding author on request.
